# A review of nutritional recommendations for scuba divers

**DOI:** 10.1080/15502783.2024.2402386

**Published:** 2024-09-24

**Authors:** Rhiannon J. Brenner, Kiran A. Balan, Marie P. L. Andersen, Emmanuel Dugrenot, Xavier C. E. Vrijdag, Hanna Van Waart, Frauke Tillmans

**Affiliations:** a Divers Alert Network, Research, Durham, NC, USA; b University of Brest, ORPHY’s Laboratory, Brest, France; c The University of North Carolina at Chapel Hill, Gillings School of Public Health, Chapel Hill, NC, USA; d The University of North Carolina at Chapel Hill, Department of Biomedical Engineering, Chapel Hill, NC, USA; e The University of Auckland, Department of Anaesthesiology, Auckland, New Zealand

**Keywords:** Health, nutrition, scuba diving, physiology, pressure, metabolism

## Abstract

**Background:**

Scuba diving is an increasingly popular activity that involves the use of specialized equipment and compressed air to breathe underwater. Scuba divers are subject to the physiological consequences of being immersed in a high-pressure environment, including, but not limited to, increased work of breathing and kinetic energy expenditure, decreased fluid absorption, and alteration of metabolism. Individual response to these environmental stressors may result in a differential risk of decompression sickness, a condition thought to result from excess nitrogen bubbles forming in a diver’s tissues. While the mechanisms of decompression sickness are still largely unknown, it has been postulated that this response may further be influenced by the diver’s health status. Nutritional intake has direct relevancy to inflammation status and oxidative stress resistance, both of which have been associated with increased decompression stress. While nutritional recommendations have been determined for saturation divers, these recommendations are likely overly robust for recreational divers, considering that the differences in time spent under pressure and the maximum depth could result nonequivalent energetic demands. Specific recommendations for recreational divers remain largely undefined.

**Methods:**

This narrative review will summarize existing nutritional recommendations and their justification for recreational divers, as well as identify gaps in research regarding connections between nutritional intake and the health and safety of divers.

**Results:**

Following recommendations made by the Institute of Medicine and the Naval Medical Research Institute of Bethesda, recreational divers are advised to consume ~170–210 kJ·kg^−1^ (40–50 kcal·kg^−1^) body mass, depending on their workload underwater, in a day consisting of 3 hours’ worth of diving above 46 msw. Recommendations for macronutrient distribution for divers are to derive 50% of joules from carbohydrates and less than 30% of joules from fat. Protein consumption is recommended to reach a minimum of 1 g of protein·kg^−1^ of body mass a day to mitigate loss of appetite while meeting energetic requirements. All divers should take special care to hydrate themselves with an absolute minimum of 500 ml of fluid per hour for any dive longer than 3 hours, with more recent studies finding 0.69 liters of water two hours prior to diving is most effective to minimize bubble loads. While there is evidence that specialized diets may have specific applications in commercial or military diving, they are not advisable for the general recreational diving population considering the often extreme nature of these diets, and the lack of research on their effectiveness on a recreational diving population.

**Conclusions:**

Established recommendations do not account for changes in temperature, scuba equipment, depth, dive time, work of breathing, breathing gas mix, or individual variation in metabolism. Individual recommendations may be more accurate when accounting for basal metabolic rate and physical activity outside of diving. However, more research is needed to validate these estimates against variation in dive profile and diver demographics.

## 1. Introduction

Scuba diving is an increasingly popular activity that involves the use of specialized equipment and compressed air to breathe underwater. While incidents such as decompression illness are generally rare events, occurring approximately only every 4 in 10,000 recreational exposures, it is still considered an inherently risky sport due to the associated exposure to a pressurized environment [1]. While breathing compressed air at depth, a diver’s tissues become saturated with inert gas over time. As divers ascend from depth, inert gas begins to come out of solution, often forming venous gas emboli, which can be detected via doppler audio recordings or using 3D ultrasound technology. While venous gas emboli alone do not necessarily pose a threat to the diver, they do act as an indicator of “decompression stress,” or relative risk of decompression sickness [2]. While the exact mechanism(s) of decompression sickness are still largely unknown, connections have been drawn between elevated decompression stress, vascular dysfunction, and inflammatory response [3,4]. This correlation has been established by Wang et al. who observed a difference in the suppression of diving-induced endothelial reactive oxygen species (highly reactive molecules that drive oxidative stress) based on the bioavailability of endogenous (innately produced) antioxidants that mediate endothelial cell dysfunction at depth during simulated air dives [5]. Furthermore, Wang et al. observed an elevated inflammatory state and decreased bioavailability of antioxidants in rats exhibiting decompression sickness symptoms after simulated air dives [6]. It has been previously demonstrated in humans that changes in vascular activity and immune response can be mediated by diet and that undernutrition may impair homeostatic and cognitive functions [7–10]. Considering the inherent increase in energetic demand that takes place under pressure [11,12], and the increased energy requirement that comes from thermal conduction and water resistance [13], divers may benefit from a robust or specialized diet in order to meet metabolic needs and potentially reduce risk of dive-related injury or illness. This narrative review seeks to explore the relationship between the high-pressure environment and the basic nutritional needs of scuba divers.

## 2. Energetic intake & macronutrient distribution

The body constantly balances its own energetic demands with food intake. Energy requirements are variable by a combination of demographic factors like sex, age, weight, height, and basal metabolism, as well as lifestyle factors like exercise frequency and non-exercise activity thermogenesis. Together these make up an individual’s total daily energy expenditure. If an individual’s total daily energy expenditure is greater than their energetic intake, they will lose weight, whereas if the individual’s total daily energy expenditure is less than their energetic intake, they will gain weight. While this explanation is slightly oversimplified, the described differences between total daily energy expenditure and energetic intake are known as a negative (total daily energy expenditure > intake) or positive (total daily energy expenditure < intake) energetic balance respectively. Macronutrients are the body’s major source of energy and consist of protein, carbohydrates, and fat. The energetic value of any food can be categorized by the combination of macronutrients it contains (~17 kJ or 4 kcal/g protein, ~17 kJ or 4 kcal/g carbohydrates, ~38 kJ or 9 kcal/g fat) [14]. Each macronutrient is essential for a variety of life-sustaining metabolic processes, therefore achieving adequate distributions of each macronutrient is critical for overall health. There may be unique energetic and macronutrient considerations for divers, given their exposure to high-pressure underwater environments.

### 2.1. The energetic requirements of a pressurized environment

The most comprehensive research on nutrition for divers has been undertaken in saturation diving who remain under pressure for extended periods of time. For chamber dives at depths of ~305 msw, Thorp and Doubt recommended ~14600 15,100 kJ (3500–3600 kcal) per day for each diver, with 30% of the energy provided by fat, 10–15% from protein, and 55–60% from carbohydrates [15]. Diets following these guidelines were successfully employed to meet or exceed the metabolic demands for three dives employing an unspecified heliox blend (a breathing gas that uses a mix of helium and oxygen) to ~36 msw. This diet was also employed for divers with a light to moderate workload in two, 28-day chamber dives with 15 days at ~304 msw. When energetic intake fell below Thorp and Doubt’s energy recommendations, due to decreased appetite at ~304 msw, divers demonstrated net weight loss [15]. However, net weight gain would be expected for dives shallower than ~90 msw if Thorp and Doubt’s nutritional guidelines were followed, due to the decreased metabolic demand and a potential attenuation of the appetite decrease effect seen at ~304 msw [15]. Because all data by Thorp and Doubt was collected within chambers, where the impact of the aquatic environment on physiological function is absent, there is potential for a difference in energetic need between open water and chamber dives. Past research has implied that the greater the depth, the greater the increase in physiological demand for the diver, especially regarding cardiovascular, urinary, and respiratory functions due to the inherent detriment of pressure on circulation and fluid dynamics [16].

Busch-Stockfisch and Böhlen reported a higher recommended range of energetic requirement at 13,315–15,973 kJ (~3490–3920 kcal) for working divers breathing an unspecified trimix blend (a breathing gas containing oxygen, nitrogen, and helium) between 200 and 600 msw [17]. Their diet recommendations were developed by integrating standard nutritional guidelines with estimations of metabolic demands for active work (840 kJ or ~201 kcal·hour^−1^) and standby (157 kJ or ~38 kcal·hour^−1^). They then examined the energetic output of 3–4 divers between 26 and 42 years old on 6 simulated working trimix dives [17]. The influence of pressure on energy metabolism was found to remain relatively small at 200 msw, but energy requirements rose disproportionately between 200 and 360 msw. For working conditions below 450 msw, energy requirements increased ~30% relative to the expected total energy needs at surface level [17]. An overall decrease in body weight was found during the first 10–20 days for all dives and following the 20th day for all dives below 250 msw. The mean loss in muscle mass was 1.98 kg and the mean loss in total body mass was 2.65 kg over the course of all saturation dives. While it is possible some of this may be attributed to a loss of movement within the confined quarters of the chamber, muscle loss is still notable considering the welding work undertaken by divers burned ~837 kJ·hour^−1^ (200 kcal·hour^−1^). No influence on the rate of change to body weight and body fat was found from diving depth, duration of the dive, or the proportion of working days. In all dives except the shallowest, the overall muscle mass of divers decreased in all divers but two. The amount of time that divers spent working was found to be the most essential factor in the maintenance of muscle mass [17].

Contrary to the temperature-controlled environment of saturation diving, recreational divers experience significant heat loss primarily via conduction when immersed due to the high heat capacity of water, which yields heat loss rates 20–27 times greater than air, when not accounting for thermal insultation provided by a dive suit [18]. Heat loss initiates compensatory action from the body, increasing metabolic rate and energy demands to maintain a safe internal body temperature. Heat loss, and therefore energetic demands, would be exacerbated in diving conditions with colder water and inadequate/ill-fitting thermal protection. The increased density of breathing gases as a function of depth also increases energy demand via the associated respiratory evaporative heat loss and increased airway resistance [11,18,19]. The use of lighter molecular weight gases like helium may not entirely resolve this either, as helium is seven times more conductive than nitrogen and increases the body’s rate of heat exchange thereby increasing energy demands.

The increase in energy requirement detected by Busch-Stockfisch and Böhlen [17], and Thorp and Doubt [15] may further be explained by a decreased rate of protein synthesis stimulating catabolism via alteration of mammalian target of rapamycin complex 1 (mTORC1) signaling [10,18]. This mechanism may not account for the disproportionate rise in energy requirements below 200 msw, but it has been highlighted by a few key studies [17,20]. When following Thorp & Doubt’s intake recommendations at ~56 msw with a chamber atmosphere of ~0.046 MPa (0.45 ATA) oxygen, a balance of helium and a maximum nitrogen partial pressure of ~0.101 MPa (1.0 ATA), a decrease in protein synthesis rate was found and estimated to be around 30% using the urea method and 40–50% using the ammonia endpoint method [20]. These are sizable drops considering most participants were found to have a positive nitrogen balance throughout the experiment and had a normal initial protein synthesis rate. Though only seven full datasets were acquired, this measured change in protein synthesis is noteworthy considering all participants were healthy, fit, young males on a diet providing adequate protein. In addition to decreased protein synthesis rate, a decreased incorporation of a radioactive nitrogen isotope (^15^N) into fibrinogen and hippurate, and a decreasing trend in the fractional synthesis rate of fibrinogen were found. This indicates that the mechanism of decreased protein synthesis may lie within acute changes in liver metabolism [20]. Observation of liver dysfunction has also been made by Ikeda et al. during a 30-day dive to 400 msw involving five male divers breathing an unspecified gas mix [21]. Further evidence of altered protein processing was found by Paciorek, who examined the nutritional status of 10 divers breathing an unspecified gas mix at 300 msw for two weeks and found indications, through negative nitrogen balance, that divers experience net protein catabolism at depth [22]. Similar to Thorp and Doubt, Paciorek also reported divers experiencing a reduction in appetite at depth despite an increased workload. However, Paciorek was able to prevent losses in body weight using a high protein-kilojoule diet and following the framework of prescribing each diver the equivalent diet of ~226 kJ·kg^−1^ (54 kcal·kg^−1^) of body weight a day [22]. Recommendations for increased protein intake suggest 1.3 g of protein per kg of body mass a day in order to achieve daily energy balance without significantly reducing diver appetite [11].

Attempts to calculate the actual energy expenditure of working saturation divers have been made by Deb et al. [19]. They determined a mean daily energy expenditure of 12,681.29 (SD 2146.39) kJ or 3030.9 (SD 513.0) kcal per day for 10 saturation divers in a chamber with oxygen pressure maintained at ~0.038 MPa (0.38 ATA) at depth or ~0.076 MPa (0.75 ATA) during bell runs (and implied but unspecified use of helium) on a commercial operation with a light workload, a living depth of 73 msw, and a maximum working depth of 81 msw. The same study reported a 13 (4)% increase in energy expenditure around 50 msw compared to the surface. When normalized for body mass, daily energy expenditure was 135.98 (SD 21.34) kJ·kg^−1^ or 32.5 (SD 5.1) kcal·kg^−1^ of body mass per day; between ~8025 and 11,012 kJ (1918–2632 kcal) for a 70 kg man. Despite most divers logging a daily energy intake of only 7846.3 (SD 2039.3) kJ or 1875.3 (SD 487.4) kcal per day throughout the measurement period, divers’ average body mass remained unchanged. This is likely a result of the relatively short study period and possible underestimation of daily energy intake [19]. Similar results were found by Seale et al., who found a significant increase in carbon dioxide production and energy expenditure in 10 divers breathing an unspecified heliox mix over the course of 10 days. This was found both in a ~ 56 msw environment, and in a higher-pressure environment of ~317 msw. Energy expenditure of divers was increased 13 (4)% at a ~ 56 msw, and 14 (4)% at ~317 msw, relative to surface level [12].

Recreational divers still experience increased rates of energy expenditure shallower than ~46 msw, and should also take care to increase their energetic intake appropriate to their level of work and conditions underwater [12,13,23]. Recommendations for non-saturation divers published in 1991 by Doubt et al. suggest energetic intake for 3 hours or more of diving should average 167 kJ·kg^−1^ (40 kcal·kg^−1^) of body mass per day when engaging in light-moderate work or exercise underwater. Divers engaging in 90 minutes or more of heavy work or exercise are recommended to consume 209 kJ·kg^−1^ (50 kcal·kg^−1^) of body mass per day; with “heavy exercise” being defined as a workloads reaching greater than 60% of an individual’s maximum aerobic capacity [13]. These recommendations do not account for changes in temperature, scuba equipment, depth, dive time, work of breathing, breathing gas mix, or individual variation in metabolism. Furthermore, these recommendations being primarily based on young, fit, American men, may be somewhat aggressive for women, children and elderly individuals, or even many non-Americans [24]. However, it may be possible to adjust for individual differences in energetic need by calculating a diver’s basal metabolic rate and subtracting the recommended range of energetic intake. This would provide an estimated range of the additional required kilojoules or Calories being recommended for the dive, as formulated below.

$$\;\;\;\;\;\;\;\;\;\;\;\;\;\;\;\;\;\;\;\;\;\;\;\;\;\;\;\;\;\;\;\;\;\;\;\;\;\;\;\;\;\;\;\;\;\;\;\;\;\;\;\;\;\;\;\;\;\;\;\;\;\;\;\;\;\;\;\;\;\;\;\;\;\;\;\;\;\;\;\;\;\;\;\;\;\;\;\;\;\;\;\;\;\;\;\;\;\;\;\;\;\;\;\;\;\;\;\;\;\;\left(167\;\mathrm{kJ}\;x\;\mathrm{Weight}\;\mathrm{in}\;\mathrm{kg}\right)-\mathrm{Basal}\;\mathrm{Metabolic}\;\mathrm{Rate}\;\text{\textbackslash{}break}\;\;\;\;\;\;\;\;\;\;\;\;\;\;\;\;\;\;\;\;\;\;\;\;\;\;\;\;\;\;\;\;\;\;\;\;\;\;\;\;\;\;\;\;\;\;\;\;\;\;=\;\mathrm{low}\;\mathrm{range}\;\mathrm{of}\;\mathrm{additional}\;\mathrm{recommended}\;\mathrm{kilojoules}\;\mathrm{per}\;3\;\mathrm{hrs}\;\mathrm{diving}$$

$$\;\;\;\;\;\;\;\;\;\;\;\;\;\;\;\;\;\;\;\;\;\;\;\;\;\;\;\;\;\;\;\;\;\;\;\;\;\;\;\;\;\;\;\;\;\;\;\;\;\;\;\;\;\;\;\;\;\;\;\;\;\;\;\;\;\;\;\;\;\;\;\;\;\;\;\;\;\;\;\;\;\;\;\;\;\;\;\;\;\;\;\;\;\;\;\;\;\;\;\;\;\;\;\;\;\;\;\;\;\;\left(209\;\mathrm{kJ}\;x\;\mathrm{Weight}\;\mathrm{in}\;\mathrm{kg}\right)-\mathrm{Basal}\;\mathrm{Metabolic}\;\mathrm{Rate}\text{\textbackslash{}break}\;\;\;\;\;\;\;\;\;\;\;\;\;\;\;\;\;\;\;\;\;\;\;\;\;\;\;\;\;\;\;\;\;\;\;\;\;\;\;\;\;\;\;\;\;\;\;\;\;\;=\mathrm{high}\;\mathrm{range}\;\mathrm{of}\;\mathrm{additional}\;\mathrm{recommended}\;\mathrm{kilojoules}\;\mathrm{per}\;3\;\mathrm{hrs}\;\mathrm{diving}$$

or

$$\;\;\;\;\;\;\;\;\;\;\;\;\;\;\;\;\;\;\;\;\;\;\;\;\;\;\;\;\;\;\;\;\;\;\;\;\;\;\;\;\;\;\;\;\;\;\;\;\;\;\;\;\;\;\;\;\;\;\;\;\;\;\;\;\;\;\;\;\;\;\;\;\;\;\;\;\;\;\;\;\;\;\;\;\;\;\;\;\;\;\;\;\;\;\;\;\;\;\;\;\;\;\;\;\;\;\;\;\;\;\left(40\mathrm{kcal}\;x\;\mathrm{Weight}\;\mathrm{in}\;\mathrm{kg}\right)-\mathrm{Basal}\;\mathrm{Metabolic}\;\mathrm{Rate}\text{\textbackslash{}break}\;\;\;\;\;\;\;\;\;\;\;\;\;\;\;\;\;\;\;\;\;\;\;\;\;\;\;\;\;\;\;\;\;\;\;\;\;\;\;\;\;\;\;\;\;\;\;=\mathrm{low}\;\mathrm{range}\;\mathrm{of}\;\mathrm{additional}\;\mathrm{recommended}\;\mathrm{Calories}\;\mathrm{per}\;3\;\mathrm{hrs}\;\mathrm{diving}$$

$$\;\;\;\;\;\;\;\;\;\;\;\;\;\;\;\;\;\;\;\;\;\;\;\;\;\;\;\;\;\;\;\;\;\;\;\;\;\;\;\;\;\;\;\;\;\;\;\;\;\;\;\;\;\;\;\;\;\;\;\;\;\;\;\;\;\;\;\;\;\;\;\;\;\;\;\;\;\;\;\;\;\;\;\;\;\;\;\;\;\;\;\;\;\;\;\;\;\;\;\;\;\;\;\;\;\;\;\;\;\;\left(50\mathrm{kcal}\;x\;\mathrm{Weight}\;\mathrm{in}\;\mathrm{kg}\right)-\mathrm{Basal}\;\mathrm{Metabolic}\;\mathrm{Rate}\text{\textbackslash{}break}\;\;\;\;\;\;\;\;\;\;\;\;\;\;\;\;\;\;\;\;\;\;\;\;\;\;\;\;\;\;\;\;\;\;\;\;\;\;\;\;\;\;\;\;\;\;\;\;\;=\mathrm{high}\;\mathrm{range}\;\mathrm{of}\;\mathrm{additional}\;\mathrm{recommended}\;\mathrm{Calories}\;\mathrm{per}\;3\;\mathrm{hrs}\;\mathrm{diving}$$

This can further be adjusted for dive time by multiplying the resulting estimate of additional required kilojoules or Calories by the ratio of time spent underwater. For example, one could adjust for a 60-minute dive by multiplying their additional required kilojoules by 0.33. Adding this back to a diver’s basal metabolic rate would result in a more personalized recommendation for total energetic intake on a given dive day.

$$\;\;\;\;\;\;\;\;\;\;\;\;\;\;\;\;\;\;\;\;\;\;\;\;\;\;\;\;\;\;\;\;\;\;\;\;\;\;\;\;\;\;\;\;\;\;\;\;\;\;\;\;\;\;\;\;\;\;\;\left(\mathrm{Additional}\;\mathrm{Required}\;\mathrm{kilojoules}\;x\;0.33\right)\;+\;\mathrm{Basal}\;\mathrm{Metabolic}\;\mathrm{Rate}\;\text{\textbackslash{}break}\;\;\;\;\;\;\;\;\;\;\;\;\;\;\;\;\;\;\;\;\;\;\;\;\;\;\;\;\;\;\;\;\;\;\;\;\;\;\;\;\;\;\;\;\;\;\;\;\;\;\;\;\;\;\;\;\;\;\;\;\;\;=\;\mathrm{Recommended}\;\mathrm{daily}\;\mathrm{kilojoule}\;\mathrm{intake}\;\mathrm{for}\;a\;60\;\mathrm{minute}\;\mathrm{dive}$$

or

$$\;\;\;\;\;\;\;\;\;\;\;\;\;\;\;\;\;\;\;\;\;\;\;\;\;\;\;\;\;\;\;\;\;\;\;\;\;\;\;\;\;\;\;\;\;\;\;\;\;\;\;\;\;\;\;\;\;\;\;\left(\mathrm{Additional}\;\mathrm{Required}\;\mathrm{Calories}\;x\;0.33\right)\;+\;\mathrm{Basal}\;\mathrm{Metabolic}\;\mathrm{Rate}\text{\textbackslash{}break}\;\;\;\;\;\;\;\;\;\;\;\;\;\;\;\;\;\;\;\;\;\;\;\;\;\;\;\;\;\;\;\;\;\;\;\;\;\;\;\;\;\;\;\;\;\;\;\;\;\;\;\;\;\;\;\;\;\;\;\;\;\;=\mathrm{Recommended}\;\mathrm{daily}\;\mathrm{Caloric}\;\mathrm{intake}\;\mathrm{for}\;a\;60\;\mathrm{minute}\;\mathrm{dive}$$

This calculation provides a more individualized recommended range of energetic intake for divers based on existing data from the Naval Medical Research Institute. The accuracy of these estimates may further be dependent on the methodology used to estimate individual basal metabolic rate and the veracity of recorded exercise activity outside of the dive itself [25,26]. Alternative attempts to estimate energy expenditure by scuba divers have been provided via indirect calorimetry by Michniewski, who calculated that a diver breathing air with a respiratory quotient of 0.85 will produce 20.29 kJ (4.85 kcals) of energy for every 1 liter of oxygen consumed [23]. This translates to an average of 22.59 kJ·min^−1^ (5.4 kcal·min^−1^) with minimal movement and 41.00 kJ·min^−1^ (9.8 kcal·min^−1^) with maximum movement in open water [23]. However, it should be noted all these dives were undertaken in dry hyperbaric chambers. Different physiological responses to diving in a wet environment, as well as shallower depths and shorter times may result in differences in energetic demand. Research is therefore needed to validate these energetic estimates in recreational divers.

### 2.2. Protein intake

Protein is crucial for repairing and strengthening muscle and bone, making it especially important during periods of reduced food intake, aging, and recovery [27]. General guidelines by the Food and Nutrition Board of the Institute of Medicine suggest that 10–35% of daily energetic intake should be derived from protein [28]. Layman suggests that protein needs should be dependent on body weight rather than a percentage of energy intake for increased accuracy, which aligns with Thorp et al.’s recommendations [13,27]. Individuals with reduced energy intake would not necessarily meet basic protein needs for metabolic functions if they were eating only 10–35% of their kilojoule intake from protein. Layman also asserts that protein need should be specific to lean tissue mass and emphasizes that an individual’s dietary protein need stays consistent despite the fluctuation in their daily energy intake [27].

While the suggested protein intake for recreational divers is 1.0 g·kg^−1^ of body weight to not reduce appetite, divers may benefit from eating more than the recommended amount [11,13]. It should be noted that the Institute of Medicine’s estimate for total protein requirement for active individuals in grams per day is higher, suggesting 1.2 to 1.8 g·kg^−1^ of body weight be consumed depending on level of physical activity. The Institute of Medicine’s higher recommendation is based on data collected from athletes who regularly participate in endurance sports [14,28]. The rate of protein synthesis has been shown to be decreased by half at 56 msw [11,29], meaning saturation divers should increase their dietary protein intake to prevent skeletal muscle mass loss. However, it is unknown whether changes in liver function and therefore protein synthesis are detectable at recreational depths, though there is a potential for oxidative stress [30].

Additionally, as someone ages, there is a decrease in the efficiency of protein utilization due to the body’s decrease in anabolic drive for lean tissue [27]. Thus, Layman emphasizes the importance of consuming more nutrient dense essential amino acids [27]. Older divers should account for their decreased protein utilization by consuming high-quality proteins rich in essential amino acids. Additionally, older divers should focus on the quantity of protein consumed by meeting their daily protein intake need. Adults require 30 g of total protein or 15 g of essential amino acids at each meal to stimulate skeletal muscle protein synthesis. Consuming dietary protein at breakfast (at least 30 g) is important to replenish body proteins after an overnight fast pushes the body into a catabolic state [27]. In adults, protein synthesis is thought to be triggered by leucine, an essential amino acid that regulates the insulin mammalian target of rapamycin (mTOR) signal pathway. This anabolic response starts after the adequate protein or leucine intake is met, meaning individuals consuming the bulk of their daily protein later in the day and in a single meal are delaying the start of protein synthesis [27]. This is due to the body’s inability to store a daily supply of protein, as it can do with carbohydrates as glycogen [31].

Foods that are considered complete proteins have all nine essential amino acids. Most animal-based proteins (beef, pork, poultry, fish, eggs, dairy) are complete proteins [32]. Total protein requirements may not necessarily increase with age, but the quality of protein becomes increasingly important [27]. If following a vegetarian or vegan diet, it is highly recommended to increase the variety and volume of protein sources [33]. Vegetarian and vegan options for complete proteins include soy, pea, buckwheat, chia, and quinoa [33].

Diving culture can be on-the-go, so packing a balanced meal could help divers meet their metabolic needs. Eating a variety of complete proteins at several meals throughout the day will help maintain lean muscle mass long term [27,32]. Although straying from these guidelines occasionally will likely not have an impact on the short term, consistent underconsumption of high-quality protein can break down muscle mass [29]. This is especially pertinent to divers who are often in innately energetically demanding high-pressure environments.

### 2.3. Carbohydrate intake and the Ketogenic Diet

Carbohydrates play an important role in the body, as they are broken down into glucose to be used as an energy source upon consumption or stored in the liver as glycogen. Most diets consist primarily of carbohydrates. The Food and Nutrition Board of the Institute of Medicine recommends 45–65% of energy be derived from carbohydrates and Doubt and colleagues suggest 50% of a non-saturation diver’s total kilojoule intake be derived from carbohydrates [13,14]. Given their importance for bodily function, a diet consisting of very few carbohydrates would appear counterintuitive; however, there is societal and research interest in a specific carbohydrate restricting strategy called the ketogenic diet. The ketogenic diet is a nutritional strategy where daily kilojoule intake is built mainly on fats and intensely limits carbohydrates to achieve a starvation state called ketosis [34]. In the state of ketosis, the body produces ketone bodies: acetoacetate (AcAc), β-hydroxybutyric acid (β-HB), and acetone. Ketone bodies are produced by the liver and used peripherally as an energy source when glucose is not readily available [35]. The ketogenic diet has been studied beginning in the 1920s as a treatment of drug-resistant epilepsy and contemporarily it has been popularized for weight loss [34–36]. The diet has also shown evidence toward the reduction of inflammation and oxidative stress which may relate to risk of decompression sickness [37,38]. Finally, *potential* anticonvulsant and neuroprotective properties, theorized to be linked to elevated ketone bodies, highlight the diet’s possible applications in the protection against central nervous system oxygen toxicity-induced seizures Central nervous system oxygen toxicity is of particular concern amongst divers breathing high partial pressures of oxygen. The exact mechanisms for the potentially neuroprotective effects of ketosis remain unknown [39].

A small observational study had three closed-circuit rebreather divers eat a ketogenic diet for four days prior to four days of 3+ hour active diving with a maximum oxygen partial pressure of ~0.152-0.162 MPa (1.5–1.6 ATA) [40]. None of the three divers reported signs or symptoms of central nervous system oxygen toxicity. The exposure to “only” 0.162 MPa (1.6 ATA) PO_2_ [41], may have been too conservative to elicit symptoms in this very small uncontrolled and underpowered study. The potential of the ketogenic diet to reduce diving-induced oxidative stress and inflammation has been tested in another group of six overweight divers [42]. The researchers reported a significant decrease in biomarkers for both lipid peroxidation (via 8-isoPGF2_α_) and inflammation (via IL-1β, IL-6, TNF_α_) when comparing a ketogenic diet group to a control group. Their findings suggested a potentially protective effect of ketosis toward some diving-induced oxidative stress and inflammatory status *when diving nitrox*. However, the data showed no significant difference when diving with air. An animal study measured oxidative stress (via plasma malondialdehyde and glutathione thiol) and inflammation (IL-1β, IL-6, TNF_α_) in rats following a simulated air dive at ~0.709 MPa for 90 minutes. Researchers reported significant decreases in biomarkers for both oxidative stress and inflammation in a ketone body-treated group compared to controls [38]. There are many possible explanations for the observed discrepancies in the reduction of biomarkers when breathing air in these two studies. Examples include obvious anatomical differences, anthropomorphic specificity (overweight divers), diver age (median 55.2), large variation in simulated dive conditions, and small human sample size (6 divers). More research is needed to understand the role of a ketogenic diet for protection against diving-induced oxidative stress and inflammation.

Ketone supplementation can be used to elevate ketone bodies levels without a diet regime, which would eliminate the obvious burden of intensely limiting carbohydrates to maintain the ketogenic diet. Three animal studies tested the utility of a synthetic ketone ester: 1,3-butanediol acetoacetate diester (BD-AcAc_2_) in diving. In two of these studies, adult rats breathed 100% oxygen at ~0.51 MPa (5.0 ATA) until the appearance of seizures. Both studies showed a later onset of seizures in rats when supplemented with the ketone ester as compared to controls [43,44]. This is in line with the epilepsy studies supporting the neuroprotective effects of ketosis [39]. Research examining the possible neuroprotective effects of ketosis for central nervous system oxygen toxicity is limited to rat models. The third animal study with BD-AcAc_2_ exposed rats to ~0.71 MPa (7.0 ATA) breathing air for 90 minutes followed by rapid decompression to induce decompression sickness [38]. The 26 rats which had ingested ketone esters had lower incidence of decompression sickness symptoms compared to 26 saline controls (34.6% vs 73.1%; *p* < 0.01). They showed no significant difference in venous gas emboli bubble counts, although the treatment group showed a decreasing trend.

The large dosages of BD-AcAc_2_ given to animals are not safely scalable or practical for human consumption. There are also observed difficulties with palatability and gastrointestinal symptoms in humans [37]. More research is needed testing new ketone ester formulations for their effective dosages, bioavailability, and safety for chronic human use. Female representation in sample selection is important for future research of ketosis given evidence of sexual dimorphism in hormone and tissue dynamics (insulin, ghrelin and TNFα) induced by the ketogenic diet [45]. Of the research discussed, all animal studies used only male rats (or failed to report sex) and only one human study contained a single female participant [38,40,42–44].

One final possible merit to the ketogenic diet is its observed utility for weight loss [32]. This could theoretically benefit overweight or obese divers by lowering body fat, benefiting overall health. However, weight loss would occur in any diet consisting of a energetic deficit and many less restrictive diets may have increased palatability and simplicity. For both human and rat models, the research regarding the ketogenic diet and diving is promising but still in its infancy. Currently, there is not enough evidence to encourage the use of the ketogenic diet or ketone supplements in divers for the protection against seizures, oxidative stress, or inflammatory status.

## 3. Micronutrient distribution

### 3.1. Antioxidants and oxidative stress

Micronutrients are vitamins and minerals that are essential to maintain and regulate a range of physiological functions. Many micronutrients function as antioxidants, neutralizing free radical oxygen and reducing oxidative stress [21,46,47]. This may be pertinent to divers due to the potential for oxidative stress to exasperate inflammation, and/or harm vascular function, in-turn, damaging physiological defenses to decompression stress [48]. Further concern of repeated or prolonged oxidative stress includes potential damage of cellular structures over time, increasing risk of chronic or degenerative disease [46]. In particular, the potential for the peroxidation of DNA is of concern due to its propensity to cause mutations [49]. Because exposure to oxygen under pressure creates the inherent potential for oxidative stress within a diver, it is worth examining the necessity of antioxidative micronutrients within a diver’s diet [5,21,30,50].

Hepatic disturbance is a concern particularly amongst saturation divers. Parenchymal cells of the liver are especially vulnerable to free radical oxygen and are highly dependent on antioxidants to maintain redox homeostasis [51,52]. Furthermore, inflammation of the liver can result in excess production of free radical oxygen, potentially resulting in cell damage and further prompting greater inflammatory response [49]. Ikeda et al. observed significant increases in plasma activity of trans-aminases and decreases in cholinesterase activity reflective of liver dysfunction in five healthy, male divers throughout the course of a 30-day dive to 400 msw with oxygen partial pressure kept at ~0.043 MPa (~0.42 ATA) during compression and at depth and increased to ~0.050 MPa (0.49 ATA) during decompression [21]. Considering the well-established connection between oxidative stress and various types of liver disease this is unsurprising [49]. Following this observation, Ikeda et al. supplemented a separate group of five healthy, male saturation divers partaking in a 40-day dive to the same depth with 600 mg of vitamin C, 150 mg of *a*-tocopherol (vitamin E), and 600 mg of tea catechins per day [21]. The divers supplemented with antioxidants exhibited no definite change in alanine aminotransferase activity, a significant reduction of aspartate aminotransferase activity, and significant increases in cholinesterase activity, indicating that it is unlikely that hepatic disturbance took place [21]. Additional monitoring of the plasma value of thiobarbituric acid and manganese superoxide dismutase (Mn-SOD) were used as markers of oxidative stress in the divers supplemented with antioxidants and did not rise, which further corroborated that oxidative stress appeared to be absent in those supplemented with vitamin C, vitamin E, tea catechins [21].

Pulmonary and vascular damage via oxidative stress is also of unique concern, considering the susceptibility of respiratory function to oxidative damage and the implications this may hold for decompression resistance [30,53]. Alterations to vascular function associated with oxidative stress have also been reported from studies focused on macro-circulation through flow mediated dilation of the brachial artery, a test of vascular function. Obad et al., for example, conducted both a non-randomized study protocol and a double-blind, placebo-controlled crossover design utilizing 2 g of vitamin C and 400 international units (representative of the bioactivity of a substance) of vitamin E as supplemental antioxidants for the non-placebo groups [54]. Dives consisted of 0.08 MPa (~0.79 ATA) oxygen for 30 minutes to a maximum depth of 30 msw. Flow-mediated dilation was assessed pre- and post-dive and a reduction in brachial artery flow-mediated dilation was detected in the placebo group, while antioxidant supplementation prevented further flow-mediated dilation reduction. Flow-mediated dilation was reduced 37% below the baseline (8.1 vs 5.1%, *p* = 0.005) 24 hours after the control dive and was found to take 72 hours to recover without antioxidant supplementation. Additionally, efforts were made to assess decompression stress via transthoracic echocardiography 30 minutes after each dive to detect venous gas emboli. While venous gas emboli were seen in all subjects, there were no significant differences in Eftedel-Brubakk bubble grades detected between control dives and antioxidant dives [54]. Conversely, Lambrechts et al. suggest that at least macro (but not micro) vascular dysfunction, could partially result from bubbles [55]. Blatteau et al. has also shown that divers presenting neurological decompression sickness symptoms also have higher mitochondrial DNA levels (*p* < 0.001), indicative of mitochondrial damage, compared to asymptomatic divers, while double stranded DNA levels are similar in both groups [56]. This goes against the bullary theory, which states that this circulating DNA comes from endothelial cells damaged by bullae, to explain the occurrence of neurological decompression sickness. The presence of this circulating mitochondrial DNA appears to show an effect of other mechanisms having an impact on the mitochondria, such as oxidative stress, and could participate in the initiation of inflammatory phenomena [57,58]. Nevertheless, a study from Wang et al. has shown that both N-acetylcysteine and vitamin C (two antioxidants) failed to prevent decompression sickness in rats following simulated air dives, while their in vitro study did confirm that N-acetylcysteine reversed the overproduction of reactive oxygen species and the attenuation of NO generation [6].

When it comes to macro and microvascular differences, Mazur et al. observed that on rat isolated vessels compressed with air, the KCl-induced contraction (which is a receptor independent contraction) was affected in the aorta after hyperbaric exposure, but not in the mesenteric artery, regardless of decompression sickness outcome [48]. This implies that that post-dive vascular dysfunction is dependent on the type of vessel. Interestingly, response to phenylephrine (which is a receptor dependent contraction) was observed in both vessels and was only impaired after mild decompression sickness, but not severe decompression sickness or no decompression sickness. This further implies that vascular dysfunction cannot be solely attributed to diving itself but is possibly influenced by circulating factors related to decompression sickness. Affected vessels may then be influenced by circulatory markers like inflammatory cytokines, which can further drive localized vascular dysfunction [59].

Further attempts to assess and manage oxidative stress response in recreational divers have been made by Yang et al. using 14 male subjects breathing compressed air on a dive to 18 msw for 47 minutes [60]. A cross-over study design was used, with one group of divers ingesting 2 g of ascorbic acid (vitamin C) daily for 6 days and the other, a placebo [60]. Oxidative stress response was evaluated using flow cytometry analysis of annexin V-positive microparticles, previously associated with oxidative stress in other studies [60,61]. Significant elevations of circulating microparticles were detected 30 minutes and 2 hours post-dive in the placebo group, while no elevation was detected in the group supplemented with ascorbic acid [60]. Additionally, divers were assessed for venous gas emboli via transthoracic echocardiography every 30 minutes for 1.5 hours post-dive. No significant difference in post-dive venous gas emboli was detected between the placebo or ascorbic acid group using a modified Brubakk grading scale [60]. The study was repeated with 10 of the 14 divers breathing a 60% O_2_ /40% N_2_ mix at atmospheric pressure for 47 minutes, yielding no elevation in microparticles for either group [60]. The results of this study reaffirm vitamin C supplementation as an effective endothelial protector under pressure, but unimpactful to venous gas emboli generation [60]. However, the lack of microparticle elevation from breathing 60% O_2_ at ambient pressure implies that oxidative stress is not responsible for microparticle generation or neutrophil activation, and that this is more likely caused by high pressure nitrogen and intrinsic hydrostatic pressure [60].

Considering multiple studies found antioxidant supplementation may prevent negative changes in physiologic function, both in recreational and saturation diving, it may benefit divers to establish an ideal minimum of exogenous antioxidants to be consumed within the period before a dive. It should be noted that the dive frequency, depth, and time are likely to play a role in the necessity of this supplementation. Oxidative stress in small doses may actually prove beneficial, as Sureda et al. observed antioxidant activation in plasma and erythrocytes but a lack of cellular damage in 7 male divers following a 25-minute dive to a maximum depth of 40 msw breathing air [30,62,63]. Blood samples were taken 3 hours post-dive, and divers exhibited an increase of plasma superoxide dismutase, erythrocyte glutathione peroxidase, and catalase activity while markers of oxidative damage, malondialdehyde and protein carbonyl derivatives, remained unchanged in both erythrocytes and plasma. This exhibits a potential for training adaptation in divers, where increased free radical oxygen may lead to angiogenesis, mitochondria biogenesis, muscle hypertrophy, and future resistance to oxidative stress without compromising vascular health [30,64]. However, this is in direct contrast to findings made by Sureda et al. on a 35-minute dive to 50 msw using air, in which 9 male divers exhibited a significant (*p* < 0.05) increase in plasma malondialdehyde 3 hours following the dive alongside similar markers of antioxidant activation [62]. It is unclear whether dive time, depth, or discrepancy in protocol is to fault for the change in results [30]. Shopov and Yordanova similarly detected a significant (*p* < 0.05) increase in oxidative stress detected via assessment of derivatives of reactive oxygen metabolites following 35 minutes at a depth of 42 m and 67 minutes of decompression utilizing an unspecified gas mix. Shopov and Yordanova also detected a reduction in antioxidant defense immediately following the dive [50]. However, neither serum antioxidant potential nor derivatives of reactive oxygen metabolites fell outside of normal levels, indicating that any oxidative stress introduced as a result of the dive will likely be reversed by preexisting antioxidant defenses [50]. It should be noted that the applicability of these results to recreational diving is limited because the study was carried out in the temperature-controlled and calm environment of a hyperbaric chamber, rather than the colder and somewhat unpredictable environment of an open-water dive [50]. Regardless, future research is needed to determine the limits and mitigation of oxidative damage.

Perovic et al. bring to attention a lack of consistency across studies that attempt to assess oxidative stress in divers due, in part, to the wide array of biomarkers with potential connections to free radical oxygen production and vascular function [30]. While covering the many biological markers that may be used for oxidative stress is beyond the scope of this review, it is important to acknowledge this inconsistency throughout dive research. Future inquiries into oxidative stress and antioxidant need in divers would benefit from agreement in methodology or an otherwise more comprehensive approach to assessing oxidative stress response.

Deb et al. suggest special care to ensure saturation divers get adequate levels of vitamin B12 and folate to facilitate red blood cell production [11]. Hemoglobin concentration has consistently been shown to decline in saturation divers, likely as a result of impaired red blood cell production consequential of extended time in a hyperoxic environment [19,65]. However, the effects of recreational diving on hematological parameters are less pronounced. Cialoni et al. report a lack of significant difference in pre- and post-dive red blood cell count and could find no relation of red blood cell count to bubble scores following a 42 meter pool dive for 40 minutes using an unspecified breathing gas [66]. Perovic et al. reports no difference following an air dive to 30 msw in red blood cell count immediately post-dive, but a statistically significant (−2.6%, *p* < 0.001) decrease 3 hours post-dive, and (−2.9%, *p* < 0.001) 6 hours post-dive [67]. Folate supplementation in divers to the recommended dietary allowance of 440 μg/day may be warranted to facilitate erythropoietin activity in saturation divers, though total intake should not exceed 1000 μg/day [11]. Conversely, iron accumulation has been reported in saturation divers, increasing their risk of type 2 diabetes, warranting a limit of iron intake to the maximum recommended amount of 18 mg/day [11,68]. Though there is little research to be found on the topic, there is no current evidence that recreational divers necessarily require additional intake of B12 or folate beyond the standard recommended daily intake for their individual demographic. Similarly, iron accumulation has not been detected in recreational diving populations to the author’s knowledge.

Because of the extended time saturation divers spend away from sunlight, vitamin D supplementation is also in order to maintain serum 25-hydroxy vitamin D above 50 nmol/L, the current threshold recommended by the Institute of Medicine [11]. Deb et al. recommends saturation divers be supplemented with a minimum of 2000 and up to 4000 international units of vitamin D daily, depending on the length, location and season of the dive [11]. However, this is unlikely to be of concern for recreational divers, who often spend time outside before and after diving, and are underwater for a minimal amount of time. The Institute of Medicine recommends that anyone between the ages of 1 to 70 get an average of 15 mcg of vitamin D daily [69]. With this said, older adults – and therefore older divers specifically – are at higher risk for vitamin D deficiency due to the reduced production capacity of the skin at that age. Thus, vitamin D supplementation through food fortification or pharmaceutical supplements may be beneficial for this diving population [70].

The general recommendation of including a varied diet high in fruit and vegetables is advisable for divers in order to provide sufficient micronutrients. At minimum, divers should ensure they reach the daily recommended minimum of vitamin C and E as an exogenous source of antioxidants [11,21]. The Institute of Medicine’s recommended dietary allowance for vitamin C being 90 mg/day for adult men or 75 mg/day for adult women, and for vitamin E being 15 mg/day for all adults. Recommendations for antioxidant supplementation for saturation divers include maintaining the Institute of Medicine’s recommended dietary allowances of selenium at 55 mg/day, and zinc at 11 mg/day for men and 8 mg/day for women [19,71].

Another common concern for recreational divers is muscle cramping. While the mechanism of cramping is not well understood, it is postulated that dehydration and electrolyte imbalance are likely contributors [72,73]. Lau et al. further used a cross-over study design to compare the threshold frequency of electrically induced muscle cramps before and after downhill running in athletes who were given either 600–1200 ml (depending on their dehydration condition) of commercially available water, or OS-1 (a solution containing 2970 mg/L of sodium, 794 mg/L of potassium, 25 mg/L of magnesium, 1801 mg/L of chloride, and 18,300 mg/L of glucose) [74]. It was found that minimum stimulation (threshold) frequency for muscle cramping 30 minutes post-exercise significantly decreased following water intake, but was significantly increased following OS-1 intake, suggesting that the retention of electrolytes decreases cramp susceptibility. Considering the frequency of cramping in open water divers, and that increased loss of electrolytes via natriuresis (sodium excretion), calcium excretion, and potassium secretion have been reported at depth [16], it is advisable for divers to meet the Institute of Medicine’s minimal requirements for electrolyte intake specific to their age and sex in order to keep up with metabolic loss, especially when increased exertion is expected [71].

## 4. Hydration

Hydration is, perhaps, the most extensively researched sector of nutrition amongst recreational divers. This is likely due to the dire consequences of dehydration on the effects of decompression stress, exhibited by a significant increase in overall risk of severe decompression sickness and death in dehydrated swine [75]. This has been contradicted by Skogland et al., who found no significant differences in post-dive venous gas emboli following a 16-hour saturation dive to ~0.51 MPa (~5.0 ATA) breathing heliox in regularly hydrated rats versus rats that had been deprived of water for 48 hours [76]. However, this study is limited by the small number of rats used, and the potential mitigation of venous gas emboli generation by the use of heliox [30,77]. Furthermore, the use of venous gas emboli grades as a sole marker of decompression stress remains controversial [77]. A more recent study conducted by Wang et al. on a larger population of rats during a simulated air dive supports the notion that pre-hydration can reduce decompression sickness-occurrence [78]. Pre-dive hydration reduced the proportion of severe decompression sickness from 47% with no hydration, to 29% with low hydration (defined by intra-peritoneal injections of NaCl 0.9% 0.1 ml·100 g^−1^ body mass at 24 hours, 12 hours, and 30 minutes prior to the dive), and 0% with high hydration (intra-peritoneal injections of NaCl 0.9% 1 ml·100 g^−1^ body mass, following the same timeline). Wang et al.’s study further saw an increased proportion of rats without any signs of decompression sickness from 40% in the group with no hydration, to 57% with low hydration, and 93% with high hydration (Chi^2^ *p* = 0.041) [78].

Pre-hydration as a mitigator of decompression sickness risk has also been studied in humans by Gempp et al. using precordial pulsed doppler and venous gas emboli grading via the Spencer scale converted to Kissman Integrated Severity Score (KISS) [79]. Gempp et al. examined 8 healthy military divers in a crossover trial in which divers were provided with either no pre-dive hydration 90-minutes, or 1300 ml of a saline-glucose beverage containing 157 milliequivalents per liter of sodium (Na+) and 23 g·l^−1^ carbohydrate to be drank within 50–60 minutes prior to a 30-min air dive to 30 msw. Divers provided blood and urine samples prior to and 60 minutes following each open water dive to a maximum depth of 30 msw for 30 minutes, with a decompression rate of 15 msw·min^‑1^ and a 9 min stop at 3 msw. Overall, post-dive KISS grades were significantly lower with pre-hydration than without (mean KISS 3.5 vs 19.4, *p* = 0.031), though one diver did display a slight increase in venous gas emboli grade with pre-hydration versus the control. Post-dive differences in plasma volume were highly significant, with a mean increase of 3.5% seen following fluid ingestion and a return to baseline post-dive, versus a post-dive decrease of 2.2% without fluid ingestion. No significant differences in plasma surface tension were detected before and after fluid intake or post-dive for either protocol, though a significant difference in baseline was displayed between protocols. Pre-hydration was found to have significantly attenuated both post-dive weight loss and negative water balance in comparison to results without pre-hydration. Though limited by the low number of participants, this study supports a connection between pre-dive hydration and reduced decompression stress in humans.

Regardless of decompression risk, special consideration should be given to water intake for divers to prevent dehydration; considering potential subjection to other dehydrants such as prolonged sun exposure, breathing dry air, extended immersion in warm saltwater, seasickness, caffeine/alcohol consumption, and long bouts of travel [80–82]. Furthermore, divers may be subject to a phenomenon known as immersion diuresis, an increase in urine production and decrease in water absorption as a result of vasoconstriction following exposure to water [16]. The original recommendation provided by Doubt et al. is that divers consume an absolute minimum of 500 ml of fluid per hour for any dive longer than 3 hours [13]. This estimate for minimum fluid ingestion is supported by estimates of decompression stress relative to fluid intake made by Han et al. This study found a significant decrease in post-dive Spencer bubble grades amongst divers consuming 0.69 liters of water two hours prior to diving versus those that drank none prior to a 25-minute dive to 30 msw using an unspecified breathing gas [83]. It should be noted, however, that overhydration is also a concern for divers. Divers who consumed 2.3 liters of water prior to diving showed similar bubble grades to those who drank no water, potentially due to a positive correlation between water retention and bubble retention [83]. Though the connection between bubble formation and serum surface tension is somewhat contradicted by the aforementioned results of Gempp et al., it cannot be entirely discounted considering that existing data has not been extensive enough to make up for the extreme variability seen in human serum surface tension [79,84]. Overhydration has also been reported in a few case studies to pose additional risk of immersion pulmonary edema; a sudden buildup of fluid in lungs known to occasionally occur in otherwise healthy individuals while swimming or diving [85,86]. Despite these findings, the risk of overhydration has yet to be adequately studied in divers and there is a lack of clear evidence to support a direct connection with immersion pulmonary edema.

Considering the risk dehydration poses to decompression sickness susceptibility, and the prevalence of immersion diuresis making overhydration a minimal risk for up to 120 minutes of submersion, it is most advisable that recreational divers focus on meeting base hydration recommendations [81,87]. The Institute of Medicine defines adequate intake of water to be 2.7 L a day for adult women, and 3.7 L a day for adult men, and recommends distribution of water intake simply be based off thirst for a majority of the population [88]. However, it is specifically noted that certain groups, including athletes, individuals that are ill, individuals in hot environments, and the elderly, cannot solely rely on their sense of thirst and may need to drink beyond the defined adequate intake. While the Institute of Medicine does not officially designate an upper intake limit for water, estimates of the maximum excretion rate of a healthy adult kidney fall between 0.8 and 1.0 L per an hour, or 20 L a day [89]. Based off current research, the existing recommendation made by Doubt et al. of drinking 500 ml of water/hour when diving longer than 3 hours are well within reason.

## 5. Conclusion

Because preexisting nutritional recommendations for divers were initially based on data collection from saturation and military diving, they may be outdated or somewhat excessive for the average recreational diver. However, pairing extrapolations from previous nutritional recommendations with the guidelines set by the Institute of Medicine may prove a more effective way to estimate diver’s metabolic needs. Energetic need can be estimated with higher accuracy by accounting for individual metabolism and differences in dive time.

Recreational divers are recommended to consume 167–209 kJ·kg^−1^ (40–50 kcal·kg^−1^) of body mass, depending on their workload underwater, in a day consisting of 3 hours’ worth of diving shallower than ~46 msw. Recommendations for macronutrient distribution mostly fall within the range of guidelines made by the Institute of Medicine, where 50% of intake is derived from carbohydrates, and less than 30% of intake is derived from fat [13,28]. Based on animal studies ketogenic diet and ketone bodies could potentially protect against seizures, oxidative stress, and/or increased inflammation. However, carbohydrate restriction, the ketogenic diet, and/or ketone supplementation is not currently recommended to divers. Recreational divers are recommended to consume 1 g of protein/kg of body mass a day to mitigate loss of appetite and meet energetic requirements, lower than the Institute of Medicine’s recommendation of 1.2–1.8 g/kg of body mass daily [13,28]. Older divers especially should focus on consuming 30 g of dietary protein at more than one meal throughout the day, or 15 g of essential amino acids. Special focus should consume complete proteins that have all the essential amino acids.

All divers should take special care to hydrate themselves with an absolute minimum of 500 ml of fluid per hour when diving for more than 3 hours, with more recent studies finding 0.69 liters of water two hours prior to diving is most effective to minimize bubble loads [13,83].

Existing nutritional recommendations do not account for individual differences in metabolism, changes in depth, temperature variation, or differential breathing/environmental gas mixes. Additionally, it should be noted that many recommendations are based solely off animal research or have a limited number of human participants. In such samples, there is an evident bias toward young males, not reflective of the growing female diving population, nor the many aging divers who have continued to enjoy the sport in their retirement. Many nutritional studies consist of participants recruited from a military or professional organization which may require the maintenance of a minimum level of physical fitness, potentially resulting in a skew of data. All blanket recommendations for divers should therefore be treated as a rough estimate of overall nutritional need and should not be used to initiate a specific diet plan for any individual outside of an experimental setting. Research is needed to determine whether existing nutritional recommendations are effective amongst a recreational diving population, and attempts should be made to account for differences in dive profiles.
